# Local Effects of Nest-Boxes for Avian Predators over Common Vole Abundance during a Mid-Density Outbreak

**DOI:** 10.3390/life13101963

**Published:** 2023-09-26

**Authors:** Daniel Jareño, Alfonso Paz Luna, Javier Viñuela

**Affiliations:** 1 Instituto de Investigación en Recursos Cinegéticos, IREC (CSIC–UCLM–JCCM), Ronda de Toledo 12, 13071 Ciudad Real, Spainjavier.vinuela@uclm.es (J.V.); 2 Grupo de Rehabilitación de la Fauna Autóctona y su Hábitat (GREFA), Apdo 11, Majadahonda, 28220 Madrid, Spain

**Keywords:** biological pest control, rodent outbreak, agro-environments, predation pressure, prey abundance

## Abstract

At the end of the 20th century, the common vole (*Microtus arvalis*) colonized the practical totality of agricultural ecosystems in the northern sub-plateau of the Iberian Peninsula. To prevent crop damage, chemical control campaigns using anticoagulant rodenticides have been employed. This approach has a high environmental impact, and it has been banned in most countries in the European Union, including Spain. It is therefore essential to analyze alternative methods with lower environmental impacts. Here we explored the efficacy of biological control by avian predators to reduce vole abundance by providing nest-boxes in croplands. We used an indirect index based on the presence/absence of vole activity signs to measure the effect of nest-boxes on common vole abundance. We found that vole abundance was significantly lower near occupied nest-boxes at distances less than 180 m, where vole abundance increases progressively with increasing distance to the nearest nest-box. We also observed that the predatory pressure negatively affects the vole abundance at the end of the breeding period, considering the total number of fledglings. However, the effect of nest-boxes was highly variable depending on the study area and more limited in alfalfa fields, the optimal habitat for voles in agrarian ecosystems. Thus, nest-box supplementation would be a feasible measure for the biological control of the common vole in Mediterranean ecosystems, but it needs improvements for vole control in alfalfa fields within an integrated pest control program. We provide several recommendations to improve the performance of biological control in alfalfa fields.

## 1. Introduction

In agroecosystems worldwide, rodents produce important economic losses due to the fall in crop productivity because of agricultural damage and also to the increase in production costs induced by pest treatment expenses [1,2,3]. Rodents are also associated with the transmission of several diseases that can affect public health, particularly during population outbreaks [4,5,6,7,8]. For these reasons, rodents are the direct target of intensive control campaigns that aim to reduce economic and/or public health damages [1,2]. During the last half-century, anticoagulant rodenticides (ARs) have been used in croplands and farmlands to reduce crop damage [9]. However, these pesticides can negatively affect non-target species, including protected and game species, by primary or secondary poisoning [10,11,12,13,14]. The conflict is particularly serious when extensive treatments involve second-generation ARs (SGARs), which may cause declines in the populations of vole predators, thus worsening the problem in the long term [14,15,16,17].

In Europe, there are several microtine species whose high-density phases can produce damage to croplands, especially the common vole (*Microtus arvalis*). This type of vole is a grassland species adapted to steppe habitats [18]. In European agricultural ecosystems, the common vole is particularly abundant in multiannual and perennial crops such as alfalfa, considered an optimal habitat for a grassland species adapted to steppe habitats [18,19,20], where specific annual weather conditions and landscape configuration can induce these outbreaks [21,22], especially in highly deforested areas [23]. At the same time, and particularly during high-density phases, the common vole is the main prey of several predators. This can lead to heated conflicts among farmers, local administrations, and conservationists whenever ARs are used extensively causing mortality among non-target species [24,25], and thus inducing a lack of consensus on the optimal management strategy of the vole populations [26]. The common vole is also a reservoir or spill-over agent for different zoonoses in our study area, particularly tularemia [5,27]. During population outbreaks, the inter-specific transmission of zoonotic pathogens can increase, resulting in human disease peaks [8] that could even be amplified by the use of ARs as a control method [5].

Sustainable methods to reduce the effects of rodent population outbreaks should (1) minimize negative environmental impacts, (2) increase agricultural net profit (by reducing costs of pest control and/or crop damage), and (3) reduce health risks. “Ecologically based management of rodent pests” (EBMRP) is a lower environmental impact method that takes into account ecological knowledge of the pest species [28,29]. These types of techniques are also aligned with the ongoing trend in the European Union to reduce the widespread use of chemical substances that may be a hazard to the environment or human health [30], i.e., anticoagulant rodenticides for plant protection are no longer allowed in most European countries [31].

Artificially increasing raptor abundance by providing nest-boxes or perching poles has been suggested as a biological control method in highly deforested ecosystems during the last decades in different countries as an ecofriendly alternative technique to reduce vole abundance [17,29,32,33,34,35,36]. The provision of nest-boxes facilitates the nesting of cavity raptor species predating voles, such as the common kestrel (*Falco tinnunculus*) [37] or the barn owl (*Tyto alba*) [38]. Both species could provide farmers with a low-cost ecosystem service for vertebrate pest control [17,36,39]. However, the efficacy of biological control to manage rodent populations is highly variable (from good performance to irrelevant effect) for reasons poorly known, and the technique has been poorly tested from a scientific point of view [17,40].

In this study, we analyze the local effects of a biological control experiment to manage vole populations in an area of northwest Spain, where voles have caused significant crop damage and sanitary problems in recent decades [23]. We test the effect of the supplementation of nest-boxes for two species of raptors, the common kestrel and the barn owl, on common vole abundance. Our study area is a highly deforested agroecosystem characterized by a scarcity of natural nesting sites for avian predators. Our main objective is to test if avian predators can reduce the abundance of common vole at the local scale. Our main prediction is that the common vole abundance should be lower closer to nest-boxes due to the removal of prey by predators attracted to the nest-box (especially if the nest-boxes are used by breeding raptors but also as perches by foraging raptors [36]).

Further, our secondary objective is to analyze how common vole abundance varies as a function of distance to nest-boxes and determine the scale at which the biological control is effective, with the final aim of optimizing the design of nest-box implementation to maximize its effect on vole abundance. Finally, we have as a third partial objective to analyze whether the number of total fledglings affects the abundance of voles at the end of the breeding season, considering several distance buffers.

## 2. Materials and Methods

### 2.1. Study Areas

**Figure 1:**
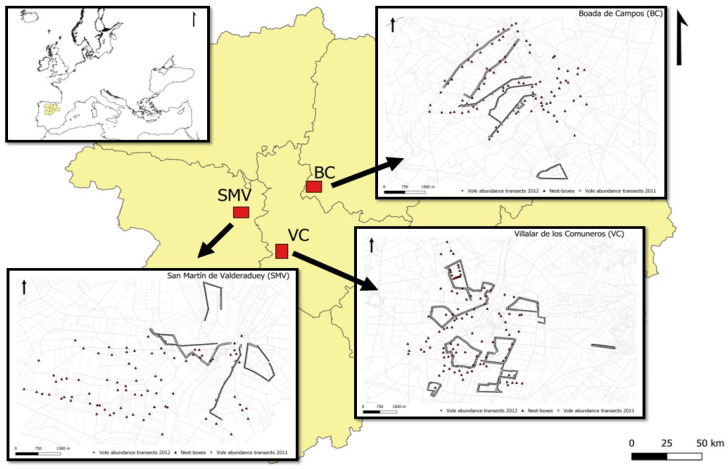
Location of the three experimental areas with nest-boxes for raptors in Castilla y León, NW Spain (red squares). Abbreviations for study areas are used throughout the text. Within the maps of every area, nest-boxes are shown as red triangles, and thick lines mark transects with sampling quadrats of vole presence. Thin lines mark crop field edges. See Figures S1–S3 in Supplementary Materials [SM] for detailed location of quadrats.

Data were collected during 2011–2012, in the winter (raptor pre-breeding season) and summer seasons (post-breeding season), in three experimental areas of Castilla y León (northwestern Spain): Boada de Campos–Capillas (BC hereafter), San Martín de Valderaduey (SMV), and Villalar de los Comuneros (VC), each of them located in the provinces of Palencia, Zamora, and Valladolid, respectively (Figure 1). Several vole outbreaks have been recorded in these areas during recent years prior to this study, especially during 2006–2007 [23,41]. This region of Spain is characterized by a predominantly agricultural landscape where most of the surface is dedicated to agrarian use (40–71% depending on provinces [42]), dominated by cereal and alfalfa crops, most as dry crops (except in VC, where irrigated alfalfa was the rule), in open homogeneous landscapes, strongly deforested, and where natural vegetation is almost limited to linear habitats as road ditches and field edges.

### 2.2. Biological Control Experiment

We installed a hundred nest-boxes in the experimental area on a surface of 2000 ha (one nest-box for every 20 ha, approximately; Figure 1) during 2009 and 2010. For each study area, we distributed the nest-boxes spatially according to their type. About half of the experimental area contained kestrel-type nest-boxes, while the other half contained barn owl-type nest-boxes. The same distribution method was used in the three study areas to increase raptor breeding populations and study the potential of using avian predators as biological controls for common vole outbreaks [36]. However, kestrels occupied both types of nest-boxes, while barn owls only used the nest-box designed for them. Thus, the final result of the experiment was that breeding kestrel populations were distributed all over the study areas, while barn owls occupied only half of the study areas.

**Figure 2:**
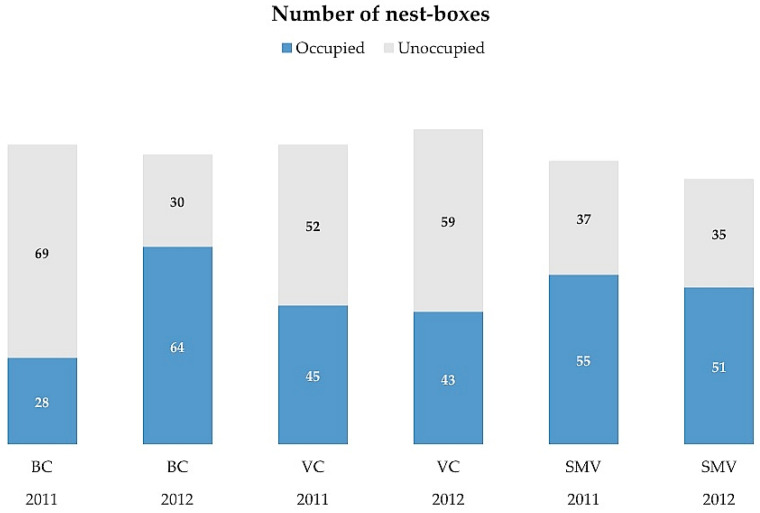
Number of nest-boxes in each experimental area per year. See Figure 1 for abbreviations of study areas.

Each nest-box was installed on a vertical pole with a height of 4.5 m. The average distance between nest-boxes was ca. 250 m (Supplementary Materials Table S1). Nest-boxes damaged during the study period were excluded from the study, so the number of nest-boxes for analyses was lower than 100 in some year/study areas. In one of the study areas (VC), a farmer installed several additional nest-boxes in 2012, which have also been included in the analysis, so the area reached a total number of 102 nest-boxes (Figure 2).

We monitored nest-boxes regularly during the breeding seasons of 2011–2012, between March and July, to identify occupied nests and to obtain reproductive parameters for each species (Table 1). Each nest-box was revised at least 3 times during the breeding season, roughly coinciding with incubation, the early nesting period, and fledging. Occupation of a nest-box by breeding kestrels or barn owls and their breeding parameters were noted for each nest-box every year, as explained in [36]. From this information, we classified each nest-box as occupied (i.e., used by a breeding pair of kestrel or barn owl in the study year, regardless of breeding success) or unoccupied (empty) (Figure 2). It is known that some of the unoccupied nest-boxes were frequently used as perching places by other common raptors in the study areas, especially the common buzzard (*Buteo buteo*) or black kites (*Milvus migrans*) [36], but we did not quantify this use of nest-boxes as perching sites by raptors.

### 2.3. Habitat

We considered six habitat types based in the type of crop or status: (1) Alfalfa (an optimal habitat for the common vole in Europe); (2) Cereal (wheat and barley); (3) Other Annual Crops (i.e., sunflower, maize, sugar beet, and peas); (4) Natural Vegetation (includes pastures and fallows); (5) Stubbles (cereals and other harvested annual crops, which in the study area may remain unploughed for several months); and (6) Ploughed (fields that have been recently ploughed and thus have no vegetation).

### 2.4. Vole Abundance

We estimated vole abundance using an indirect activity index (IAI hereafter), based on the presence/absence of fresh vole droppings and/or clippings (feeding signs) in squares of 30 by 30 cm sampled along a linear transect (Figure 1). This index has been shown to be well correlated with vole density over larger areas, and the full methodology has been described in detail elsewhere [19]. We conducted between four and eight linear transects in each experimental area during 2011 and between four and five transects during 2012 by area (see Table 2). In each transect, between 33 and 156 points were sampled approximately every 50 m. GPS coordinates for each point were taken, and each transect was repeated in February and July–August every year. The spatial distribution of transects was designed to include a representative sample of fields with the most important agrarian habitat for voles in the study area (alfalfa fields, see [19,22]) and a wide range of distances to nest-boxes. Overall, sampled points were at distances between 3–4505 m from the nearest nest-boxes (Supplementary Materials Figures S1–S3).

We used the distance to occupied and unoccupied nest-boxes as an estimator of the effect of predator activity on vole occurrence at the quadrat level, taking into account variations between years, experimental areas, and season (winter = February, and summer = August). We expected similar vole abundances near all nest-boxes in winter (as by then all nest-boxes are unoccupied for breeding, and their use as perches may be similar for all of them), while in summer we would expect lower vole abundances near occupied nest-boxes than unoccupied ones. All points that presented ploughed habitat in August were excluded from the data analysis, as the strong reduction in vole abundance caused by ploughing was the result of the destruction of burrows due to this agricultural practice [43,44].

Using the coordinates for each sampled point (the central point in every sampled square) and of each occupied and unoccupied nest-box, we used a GIS program, QGIS 3.8.3, to obtain variables describing the distance between each sampled point and: (a) the nearest unoccupied nest-box in the same year; and (b) the nearest occupied nest-box in the same year. We also used QGIS to obtain the land register identification number (hereafter “field ID”) for the fields in which each point was located from the Spanish cadastre [45]. Each sampled field had a unique ID, irrespective of the experimental area it was sampled. We also noted (during fieldwork) the habitat type of each sampling point (quadrat) within fields, because a given field could be occupied by more than one habitat type.

### 2.5. Distance to Nest-Box

We consider the distance to the nest-box, differentiating whether the nest has been occupied by a breeding pair or not, according to three distance categories, considering the radial distance that generates surfaces of 10 ha. Previous studies on the efficacy of avian predators for the biological control of rodent pests in Asia have found that one nest-box per 10 ha may significantly reduce crop damage [46]. We thus considered 3 categories of distances: A = sample points at less than 180 m from a nest-box (i.e., all points within a circle of approximately 10 ha around a nest-box); B = points between 180 m and 540 m from a nest-box (an increase in the radius equal to the previous diameter); and C = all points 540 m or further from the nearest nest-box. These three categories of distances were considered because each of them included circa 1/3 of the sampled points (being, thus, well balanced in terms of sample size).

### 2.6. Number of Fledglings

We considered the total number of raptors fledglings in the nest-boxes for two categories of distance buffers for every sample point: (1) the total number of fledglings in a circular buffer of 180 m; and (2) the total number of fledglings in a circular buffer of 540 m for every point (see Supplementary Materials Figures S4 and S5).

### 2.7. Data Analysis

We analyzed differences in abundance between the different habitat types considered in the study using an ANOVA. We study variations in the probability of vole occurrence by sampled quadrat in relation to nest-box distance using generalized linear mixed models (GLMM). We fitted the response variable (IAI per quadrat) to a binomial distribution (1 = presence of signs of recent vole activity; 0 = no presence of recent activity). We included field (agricultural plot; field ID) and habitat type as random factors in the model in order to take into account the non-independence of sample points in the same fields and habitats. We tested for differences in vole abundance (IAI) according to season (a categorical variable with two levels: winter—February; and summer—July and August), distance to nest-box (a categorical variable with three levels: A—<180 m; B—≥180 to <540 m; and C—≥540 m), year (a categorical variable with two levels: 2011 and 2012), area (a categorical variable with three levels: VC, SMV, and BC; see Figure 1), and all the interactions between them. We ran two separate analyses, one considering the distance to the nearest unoccupied nest-box and another considering the distance to the nearest occupied nest-box.

**Figure 3:**
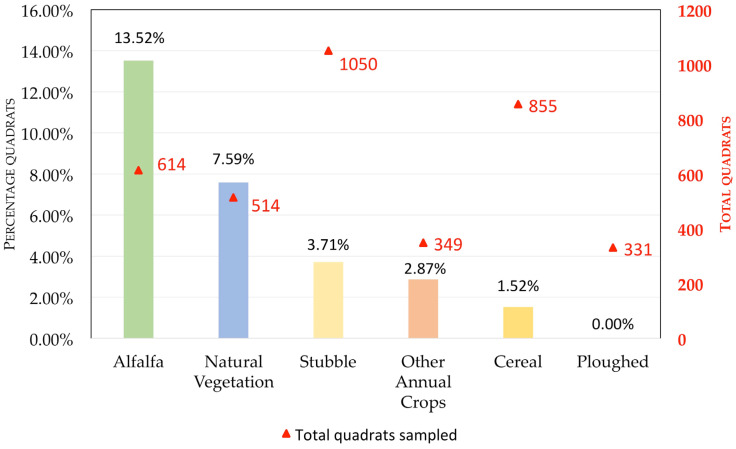
Common vole abundance (% of quadrats with presence) according to habitat type; using an indirect abundance index (IAI), based on the presence/absence of vole droppings and/or feeding signs, to measure vole abundance.

Alfalfa is an optimal habitat for common voles in agrarian ecosystems, where they reach maximum densities, serving as a refuge during low phases of population cycles and as a major demographic source for colonization of other crops during outbreaks [19,20,21,22,47,48,49,50]. Thus, it is important to assess the efficacy of nest-boxes in this particular habitat. With this aim, a GLMM analysis with the same response variable (IAI) was carried out, considering only alfalfa crops and testing the effect of the distance to the closest occupied or unoccupied nest-box, season, year, area, all their interactions, and field ID as a random factor. We also run models for the remaining habitats (excluding alfalfa) with the same procedure as the one used for the global model and described above, with comparative purposes. We did not run specific analyses for every kind of habitat because lower sample sizes (Figure 3) or a lack of representation of a given habitat in all study areas/seasons/years precluded model convergence. The results of these additional analyses are presented as Supplementary Materials and fully considered in the discussion and conclusions.

We also modeled the effect of the total number of raptor fledglings over the probability of vole occurrence by sample using GLMM, fitting the same response variable (IAI per quadrat), and considering field ID and habitat as random factors too. We tested for differences in vole abundance (IAI) just in the summer during the post-breeding season, considering also the year, study area, and their interaction. We ran two separate analyses, one considering the total number of fledglings by sample point in a buffer of 180 m and a second considering the number of fledglings by sample in a distance buffer of 540 m (see Supplementary Materials Figures S4 and S5).

Statistical analyses of the data were done using the lme4 package for R [51]. We started from the full model and selected the best model considering *p*-values for the variables, using a Type III sum of squares as the selection criterion. At each step, we dropped the variable with the highest *p*-value, stopping when all variables in the model were significant (*p*-value < 0.05). If an interaction was significant, we did not eliminate any of the components of the interaction, even if they were not significant per se.

## 3. Results

Our results showed that, as expected, the type of habitat significantly affected the index of vole abundance (F_5,3706_ = 30.86, *p* < 0.001), with alfalfa having the highest vole abundance, followed by natural vegetation, stubble, cereal, and other annual crops (that had similar, intermediate, vole abundance values), whereas recently ploughed fields had no vole presence at all (Figure 3).

**Figure 4:**
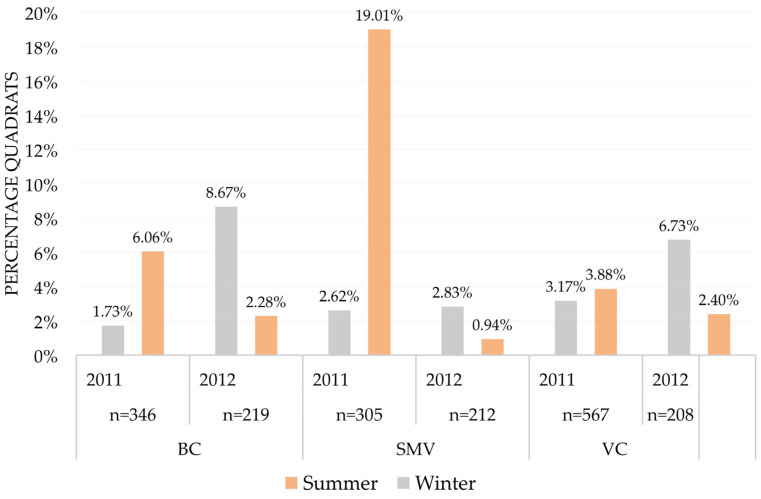
Common vole abundance (percentage quadrats with presence) in each study area considering year and season, using an indirect abundance index (IAI). Under each year appears the number of sampled quadrats per area and year (n). Abbreviations for study areas as in Figure 1.

Regarding the main factors affecting variation in vole occurrence, we found significant differences according to season, area, the interaction between year and area, the interaction between season and year, as well as the interaction between area and season (Table 3). The variation in these factors was related to the temporal variation in vole abundance that differed among our study areas and seasons. These interactions arose from the fact that there was a spatial shift in the seasonal and temporal variations in vole abundance among our study areas (Figure 4); we detected an overall increase in vole abundance from the beginning of the study, followed by a reduction. However, the time of the maximum abundance observed and its amplitude varied among areas. In the study area of Zamora, SMV, abundance peaked in the summer of 2011, strongly declining to the winter of 2012, whereas in the other two areas, abundance peaked in the winter of 2012, only declining in the summer of 2012. The variable year per se was not significant in any model (Table 3), whereas both season and area were (Table 3), with winter having an overall lower abundance than summer and SMV being the area with a higher vole abundance (Figure 4). Differences in abundance among seasons also showed variation between years, so abundance tended to be higher in the summer during 2011, but the opposite was found during 2012 (Figure 4). Finally, seasonal variation in abundance was very low in VC, very high in SMV, and intermediate in BC (Figure 4).

**Figure 5:**
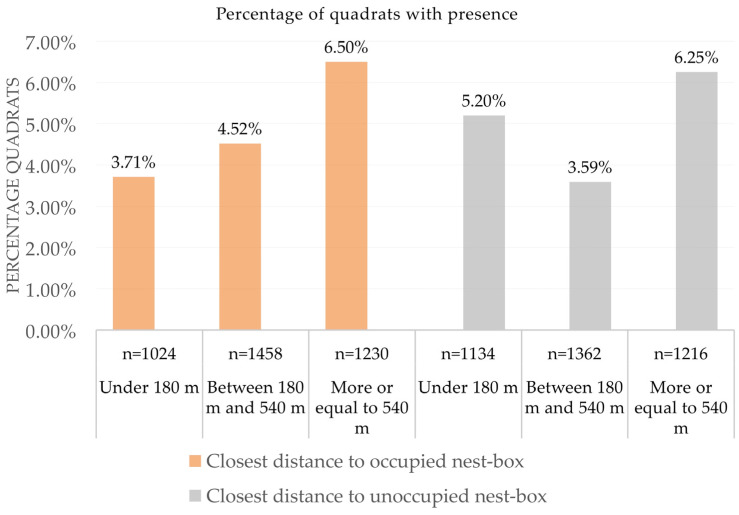
Common vole occurrence (% of quadrats with presence) according to the distance to the closest nest-box, occupied or unoccupied using an indirect abundance index (IAI). Under each column appears the total number of quadrats sampled (n).

After accounting for variations related to year, area, and season (plus habitat and field ID, included as random factors in the model), we also found that vole occurrence increased significantly when the distance to the nearest occupied nest-box also increased (Figure 5 and Table 3). However, variations in the distance to the unoccupied nest-box did not produce a significant effect on the estimated vole abundance (Figure 5 and Table 3). All second-order interactions that included distance in both models were not significant, as well as all third- and fourth-order interactions (Table 3).

The analyses considering exclusively alfalfa produced quite different results when compared to the global model (Supplementary Materials Table S2). We did not find any significant effect of distance on occupied nest-boxes or on its interactions with other factors. However, we found a significant effect of distance to unoccupied nest-boxes and its interactions with year and season (Supplementary Materials Table S2, Figures S6 and S7). Overall, the abundance of common voles in alfalfa seemed to be less affected by distance to occupied nest-boxes than in the global model, as we detected marked differences in vole presence depending on distance to occupied nest-boxes only during winter and summer 2012 in the SMV area and during winter 2012 in BC (Supplementary Materials Figure S8). However, distance to unoccupied nest-boxes had a significant effect on vole abundance in alfalfas, albeit with variation between years (lower vole abundance near unoccupied nest-boxes mainly in 2012, but not so much in 2011; Figure S6) and seasons (opposing patterns in summer and winter; Figure S7).

The analyses considering the remaining habitats, alfalfa excluded, produced results pretty similar to the global model (Supplementary Materials Table S3). We found again a significant effect of distance to occupied nest-boxes but also the interaction distance-area, so the effect of distance to nest-box was stronger and very clear in SMV and VC, but not so much in BC (Supplementary Materials Figure S9). No effect of distance on unoccupied nest-boxes was found in the global model (Supplementary Materials Table S3).

**Figure 6:**
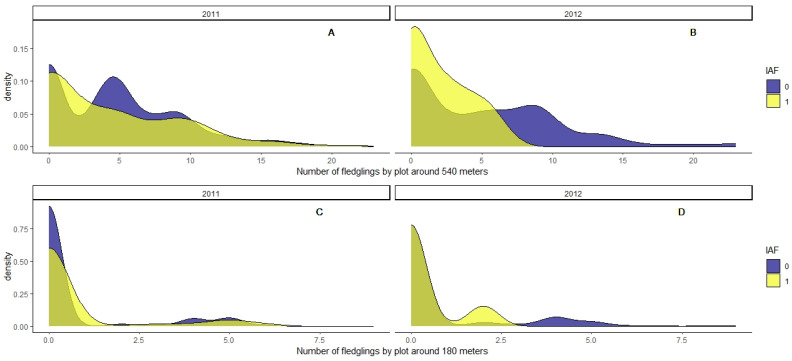
Density histogram of the number of fledglings by quadrat sample within a circular buffer of 540 m radius (**A**,**B**), or 180 m radius (**C**,**D**), according to the indirect abundance index (IAI), where “1” corresponds to recent activity or presence and “0” refers to no recent activity.

Our results showed also that the number of fledglings around 540 m for every sampled quadrat affected the probability of vole occurrence, especially during the year 2012, when the majority of quadrats with vole presence were exposed to a lower number of fledglings (Figure 6B,D and Table 4). We only found this significant effect of the nest-boxes considering the total number of fledglings per quadrat at a distance of up to 540 m (Figure 6A,B and Table 4). We found no effect of the number of fledglings on vole abundance when we modeled considering a buffer by quadrat of 180 m (Figure 6C,D and Table 4).

## 4. Discussion

Overall, our study shows that the installation of nest-boxes in highly deforested Mediterranean croplands to attract breeding pairs of avian predators can significantly reduce the abundance of common voles in crops at the local scale, near nest-boxes selected for nesting by avian predators. It is important to notice that 2011–2012 was a peak year within the cyclic demography typical of the species in the study area, with moderate maximum abundances, as compared with other recent peak years with known highest abundances (2007–2008 and 2013–2014) [23,36,41]. Thus, biological control by raptors seemed to be effective at the local level during a mid-density vole outbreak, when voles can cause more crop damage or public health problems.

The effect of distance to nest-boxes on vole abundance was detected while accounting for other variables known to affect vole numbers (study area, year, season, habitat, and field ID). Regarding the abundance of voles according to habitat, our results were consistent with results from previous studies elsewhere in Europe [18,20,47,49,52], as well as in the same study areas of Spain [19,21,22]. Thus, vole abundance was maximum in alfalfa fields, the optimal habitat. Vole abundance was smaller but still high in natural vegetation, while the rest of prospected habitats (agricultural uses) were suboptimal (Figure 3). We also found significant differences in the estimated abundance of voles among our study areas, with contrasted annual and seasonal variations (significant interactions in Table 3). The vole population peak was apparently reached earlier in SMV than in the other two study areas (Figure 4). These observed variations are surely related to different development phases of the multiannual cycle of the common vole in every study area, with a general pattern of higher vole abundance in summer than in winter (Figure 4), as reported for this species in central Europe [52]. However, in the year of the population crash (2012), abundance was higher in the winter than in the subsequent summer, due to the typical spring population crash common in decline years of rodent cyclic populations [22,41,53]. The differences in the timing of the population peak between study areas, along with the short time series of years around an outbreak, may explain why we found significant effects of area and season but only interacting effects of year with season and area in our global model (Table 3 and Figure 4). Longer-term analyses of vole cycles in our study area supported a much stronger effect of year than that of season [41]. Furthermore, in a nearby study area where the main habitat for common voles is natural pastures for livestock, it has been reported from a longer time series that seasonal variation in abundance is moderate with no statistically significant differences between spring and autumn, while year-to-year changes in vole abundance are large and highly significant [37].

With respect to differences in vole abundance and demographic patterns between study areas, VC had the more stable vole populations, with less marked peaks and lows and a general abundance relatively low when compared with maximum abundances in the other study areas (Figure 4). This fact had been previously reported [36] and confirmed in subsequent years up to the last vole population outbreak in 2019 [41]. In contrast, SMV showed the largest differences between maximum and minimum abundances (e.g., the largest peaks and lows), while in BC, an intermediate pattern was found (Figure 4). Several non-exclusive hypotheses could explain these differences in the demographic patterns between study areas (see discussion in [17]), including a potential contrast in the efficacy of biological control due to differences in management of alfalfas between study areas (see below). In fact, during the large-scale, high-density vole outbreak of 2007, VC was among the localities with the highest vole abundance and the most severe crop damage. After nest-boxes were provided, no significant crop damage was reported in VC anymore. In contrast, in BC, an area located in the middle of southern Palencia province, where more frequent, recurrent, and highest-density outbreaks have been reported [41,54], nest-boxes have pretty limited long-term efficacy and crop damages are a recurrent problem associated with vole outbreak years [55].

As we predicted, we found that the effect of the occupied nest-boxes on vole abundance increased when the distance to nest-boxes decreased (Figure 5), and this effect looked independent from several confounding variables that may affect vole abundance (no significant interactions with distance in the model, Table 3). Considering the effect of the distance to the nearest occupied nest-box, our results showed that the estimated abundance of voles at a distance of less than 180 m was approximately 18% lower than at distances between 180 and 540 m, which in turn was approximately 30% lower than at distances higher than 540 m (Figure 5). Occupied nest-boxes by breeding pairs of avian predators thus had their maximum effectiveness in an area of ca. 10 ha around them, which coincides with the results obtained with barn owls in rice fields [46] and maintains the trend found in similar studies about the efficacy of this biological control technique [17,36]. Furthermore, our results indicate a direct effect of the occupied nest-boxes that could negatively affect the vole abundance up to 90 ha around them. In contrast, we did not find any effect of unoccupied nest-boxes, which supports that breeding pairs of raptors removed voles with higher efficacy than raptors using poles of unoccupied nest-boxes as perches [36]. The detected effect of occupied nest-boxes on the estimated abundance of rodents was consistent between experimental areas, seasons, and years. We expected a higher effect of the occupied nest-boxes at the end of the breeding season, or at least dependent on the total number of fledglings (see below). Our results showed an effect of nest-boxes even at the beginning of the breeding season, which indicates that the pairs of birds of prey are already producing a negative impact on the abundances of voles during the winter, possibly due to overwintering raptors or breeding pairs staying in the nesting area all year. However, as we expected, we observed an effect of fledgling presence at the end of breeding season, with the estimated abundance of voles being lower as the number of fledglings increased, since the volume of prey removal in these local areas should be higher (see below).

**Figure 7:**
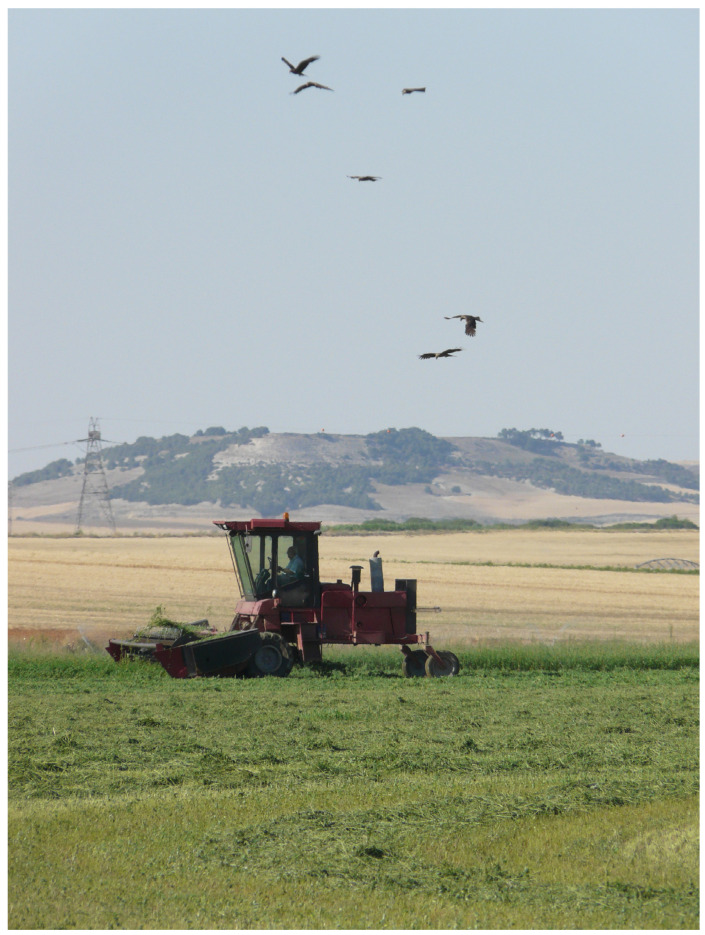
Flock of black kites searching for voles over a mowing machine in an alfalfa field of the study areas. Picture by Daniel Jareño.

When repeating the analyses considering exclusively sampling quadrats within alfalfa fields, we did not find any clear or regular effect of distance to occupied next-boxes, but an effect of distance to unoccupied nest-boxes instead, albeit more limited and irregular than what we found in the global model (significant interactions between distance to unoccupied nest-boxes and year and season) (Supplementary Materials Table S2, Figures S6–S8). Breeding kestrels may have limited access to common voles when alfalfa reaches a given cover and height, hindering the detection and capture of this prey (see [56] for a similar case with a sister species, the lesser kestrel). Supporting this hypothesis, we detected starving kestrel broods in nest-boxes at the edge of large fields with fully-grown alfalfa and high vole density. Survival and growth in these broods improved only after harvesting affected the field, allowing easy detection and hunting of voles. In fact, many aerial predator species may gather in high numbers or spend a long time foraging in recently cut alfalfa fields, preying on voles, that in these recently harvested fields become an abundant and highly accessible prey ([33] and Figure 7). However, other predators hunting more by ear than by eye, such as barn owls (or short-eared owls, *Assio flameus*, common ground breeders in the study areas during vole outbreaks [57]), could effectively hunt voles even in fully grown alfalfa, whenever good hunting perches are available, and this may explain the effect of unoccupied nest-boxes on vole abundance in alfalfa fields. Furthermore, unoccupied nest-boxes could also be used more frequently as hunting perches by other competing diurnal predators, usually expelled by breeding kestrels from the vicinity of their occupied nest-boxes, and this could also contribute to explaining the effect of unoccupied nest-boxes in alfalfas [36]. The model with the remaining habitats produced results quite similar to the global model instead (Supplementary Materials Table S3, Figures S9 and S10).

The efficacy of nest-boxes in regulating the abundance of common voles in alfalfa fields could also be modulated by the management of alfalfa. In the area where biological control has worked better (VC), alfalfas were irrigated crops, while in the other two study areas (BC and SMV), alfalfas were cultivated as a dry crop. Irrigated alfalfas can surely be more favorable for biological control, at least for two reasons: (1) Irrigation causes flooding of vole burrows, which is considered a good technique to control vole abundance [33,58], thus contributing to reducing vole numbers; (2) Irrigated alfalfa is much more productive than when cultivated in a dry regime, so the number of cuts per year can be 2–4 times higher. Thus, under irrigation, voles in alfalfa fields are easily available for aerial diurnal predators as kestrels during longer periods in the breeding season. Furthermore, farmers in VC tended to plough alfalfa fields every 3–5 years when they noticed excessive growth of vole populations within their fields. Finally, when irrigation is provided by lines of sprinklers, these unharvested lines become strips of natural vegetation used by weasels that are more commonly found within these alfalfa fields than in others without sprinklers (unpub. results). In contrast, dry alfalfa fields in the other study areas, particularly in BC, are usually maintained for longer periods (usually more than five years, reaching up to 8–10 years), which promotes a higher density of common voles during a longer period of time. Thus, different management of alfalfa in the study areas could contribute significantly to the success of the biological control program.

Overall, our results and previous research in the same study areas [19,21,22,23,55] support that the area covered by alfalfa (particularly dry alfalfa) in the agrarian landscape is the key factor causing high-density vole outbreaks, which are difficult to manage exclusively by nest-boxes. This problem is also enhanced by the fact that current agrarian fields have large size and that the main ground predator of voles, the common weasel (*Mustela nivalis*), barely hunts voles far from field edges, their main habitat in these agrarian landscapes [22,55,59]. Dry alfalfa fields are thus a sort of “paradise” for voles, providing undisturbed soil during many years for their burrows, hyperabundant high-quality food, good vegetation cover against aerial predators, and mostly the absence of their main predator within burrows.

It is important to notice that during our study, the occupancy of nest-boxes by breeding barn owls was pretty low when compared with the high occupancy rate by kestrels (Table 1) or with other similar biological control programs in other parts of the world [38,46]. Our study was developed just 2–3 years after provisioning nest-boxes in study areas where barn owls had never used this kind of nesting site before, as their nesting sites were clearly associated with human edifications or ruins in villages or the countryside. The presence of larger populations of barn owls breeding in nest-boxes could be particularly important to improve vole control in alfalfas, for the reasons argued above. Interestingly, the study area where the abundance of voles in alfalfas was apparently more reduced near occupied nest-boxes was the one where barn owls had the better occupancy rate of nest-boxes (SMV; see Table 1 and Supplementary Materials Figure S8), and the same was detected for the remaining habitats (Supplementary Materials Figure S10). Occupancy of nest-boxes by barn owls has improved in subsequent years (GREFA pers. comm.), and this may have improved the efficacy of the biological control program.

We also assessed the potential effect of the density of raptor fledglings on summer vole abundance. We found that the total number of fledglings at buffer distances of 540 m negatively affected the estimated abundance of voles, but not in the buffer of 180 m (Table 4). This can be simply explained by the higher number of fledglings present in the largest buffer, as well as the increasing mobility of these young birds as summer advances (Supplementary Materials Figures S4 and S5). The fact that we did not find a significant effect of the number of fledglings at distances of less than 180 m on the abundance of voles could also be due to a variation in the hunting range of both fledglings and pairs with respect to the size of the hunting range by adults at the beginning of the reproductive period. This variation in the hunting range may be due to a drastic reduction in the availability and accessibility of prey in crops in the summer, with most of the dryland crops already mowed and becoming stubble, a suboptimal habitat for voles with an extremely low density of individuals.

In any case, our results do not allow us to rule out the possibility of unoccupied nest-boxes used by raptors, including fledglings, as hunting perches. In our study area, the main distance between nest-boxes is around 250 m (Supplementary Materials Table S1). At this range, the effect of the occupied nest-boxes that we found might also be produced indirectly by unoccupied nest-boxes as hunting perches, particularly at distances between 180 and 540 m (Figure 5) [36]. Even this potential effect could be increased in highly deforested landscapes, as in our study area [36]. Indirect effects of predation that affect common vole behavior might also drive vole abundance, and they can be just as important as direct extraction of prey [60,61]. This indirect effect of predation and, therefore, of the nest-boxes should not be underestimated but requires additional research.

Overall, our results show that nest-boxes for avian predators locally reduce common vole populations in croplands during a mid-density population peak, thus potentially being a useful method to reduce vole abundance and mitigate crop impacts. However, during our study period, vole populations did not reach the maximum densities of other peak years, such as 2007–2008 or 2013–2014 [5,23,41]. The available information supports that when dry alfalfa covers a large part of the agrarian landscape, the efficacy of biological control in these high-density peak years is pretty limited, as it happens in southern Palencia province [22,55]. This fact is in line with the results of biological control programs in other areas, where local effects of the nest-boxes to reduce rodent populations have been found, but biological control was unable to prevent landscape-level outbreaks [62]. Thus, it is necessary to extend and improve the performance of biological control in areas where alfalfa cultivation is extended, in combination with other vole control techniques, within a framework of integrated management programs, as proposed in other agrarian areas of the world [33,63].

Finally, from an environmental perspective, it is important to note that the common kestrel, historically considered as one of the most common raptors in Spain, is currently considered as an endangered species for the first time in the history of the Spanish Red Data Books [64]. This upgrading is justified due to a drastic decline in numbers during the last 20 years, particularly in NW Spain, where massive and large-scale use of anticoagulant rodenticides in recent decades has been a main factor contributing to that deep decline [11,14,64]. Barn owls have also been affected strongly by this kind of mortality in the recent past [17,65]. Thus, the use of nest-boxes in NW Spain would not only contribute to the control of a relevant agricultural rodent pest but also promote the recovery of two predatory species that have been strongly affected in the recent past by vole control programs based on extensive use of chemical control. This can be considered a nice example of ecological restoration of a relevant ecosystem service provided by predators while ecologically compensating old-style, environmentally damaging pest control programs.

## 5. Concluding Remarks, Management Recommendations and Future Research

Our results and previous works [17,36] support the use of biological control programs based on the provisioning of nest-boxes for raptors as an effective way to control common vole pests in agricultural areas of NW Spain. However, the success of this technique is highly variable, ranging from pretty good in one study area (VC) to quite limited in another (BC).

The variation in efficacy of this kind of biological control between study areas can be largely driven by the area covered by optimal habitat for voles (alfalfa fields under dry cultivation), as well as by the management system of this crop (but see discussion in [17] for other non-exclusive hypotheses that can contribute to explaining these differences between study areas too).

To increase the global efficacy of this biological control program, it is critical to improve the occupancy rate of nest-boxes by barn owls, where necessary, by hacking programs using rehabilitated fledglings (from wildlife recovery centers) or by translocating fledglings from natural nests or occupied nest-boxes in other areas.

Biological control of vole pests, when dry alfalfa is extended in the landscape, can benefit from a number of management actions in this crop within an integrated control program, as follows:

- Promoting the use of temporal perches for raptors (easy to remove, to allow harvesting) within alfalfa fields (see [66,67] for a good example in the Czech Republic).
- Increasing the density of nest-boxes, particularly for barn owls, trying to reach the maximum density of raptors known in a similar agricultural system in Israel [34].
- Promoting any habitat management that may increase the presence of weasels as predators within alfalfa fields (i.e., leaving strips or patches of wild vegetation within alfalfa fields connected with field edges).
- Promoting the maintenance at landscape level of well-vegetated and undisturbed field edges as a refuge and source for weasels and other ground predators of voles [22,55]
- Reducing the number of years that alfalfas remain in the same field, applying deep, large-scale ploughing when vole populations in alfalfas have developed at landscape scale [68].
- Promoting actions reducing vole numbers in alfalfa fields during the non-productive season of the crop, in winter, which is also the less favorable season for survival and reproduction of voles, when minimal numbers in the yearly cycle are usually found. These actions may include temporal flooding of fields (whenever possible), but also recovering traditional management currently rare, such as using alfalfa fields as winter pastures for livestock [21,69] or removing as much possible aerial parts of alfalfa plants (leaving the field in a similar condition to ploughed fields).
- Promoting an increase in the number of cuts in alfalfa fields, as well as cuts as low as possible, when vole abundance is high. However, the use of this technique should be carefully evaluated at the local scale because it may be detrimental for endangered steppe birds that can commonly nest within this habitat, contradicting current agro-environmental measures promoting delays in alfalfa cuts to improve the population-scale productivity of those birds [50].

Although management improvements in dry alfalfa could benefit biological control of vole pests, the easiest way to reduce the impact that alfalfa fields have on the development of large-scale, high-density vole outbreaks could be by reducing the area covered by this crop at the landscape level. A reduction of >50% in the area covered by alfalfa between 1995 and 2016 in the Chezch Republic resulted in a clear reduction in the intensity of common vole outbreaks (Emil Tkadlec, pers. comm.).

With respect to future research, it should be a priority to compare the efficacy of barn owls and common kestrels as biological control agents of vole pests, particularly in alfalfa fields. Research on the sub-lethal demographic effects of increased predation pressure on vole populations is also important. We recommend also evaluating the indirect effects of the nest-boxes on other non-target species, especially birds, as done in other areas [17]. Finally, it is necessary to quantify how the reduction in vole activity produced by nest-boxes translates into increases in crop yield in our study area and whether these increases would make it economically profitable for farmers to install nest-boxes without subsidies, especially in some of the preferred habitats for the common vole, like alfalfas, as shown in a study with the levant vole (*Microtus guentheri*), a pest species that damages alfalfas in Israel [70]. Furthermore, we did not measure the possible economic benefit of biological control by avian predators, taking into consideration both the potential improvement in crop production, especially in alfalfas, and a reduction in pest control expenses (see [67] for the Czech Republic).

## Figures and Tables

**Table 1:** Reproductive parameters for the two species of raptors, common kestrel and barn owl, using nest-boxes in this study during the years 2011 and 2012. Abbreviations for study areas as in Figure 1.

Study Area	Species	Breeding Parameter	2011	2012
BC	*Falco tinnunculus*	Total clutches	27	56
		Total clutches breeding success	25	47
		Total number of fledglings	103	190
		Average fledglings by nest	4.12	4.04
	*Tyto alba*	Total clutches	1	8
		Total clutches breeding success	0	6
		Total number of fledglings	0	22
		Average fledglings by nest	NA	3.67
SMV	*Falco tinnunculus*	Total clutches	38	45
		Total clutches breeding success	32	41
		Total number of fledglings	142	168
		Average fledglings by nest	4.44	4.10
	*Tyto alba*	Total clutches	17	6
		Total clutches breeding success	14	4
		Total number of fledglings	57	11
		Average fledglings by nest	4.07	2.75
VC	*Falco tinnunculus*	Total clutches	42	38
		Total clutches breeding success	37	31
		Total number of fledglings	152	128
		Average fledglings by nest	4.11	4.13
	*Tyto alba*	Total clutches	3	5
		Total clutches breeding success	3	5
		Total number of fledglings	15	23
		Average fledglings by nest	5	4.60

**Table 2:** Number of transects in each experimental area per year, with the number of points used in the analyses (left) versus the total number of points sampled, and as percentage. The discordance between used and total numbers arise from the number of points that were ploughed in summer/autumn and were not used for analysis. “nd” stands for “not done”. Abbreviations for study areas as shown in Figure 1.

			Study Areas							
		BC			VC			SMV		
Year	Transect	Used	Total	%	Used	Total	%	Used	Total	%
2011	1	58	74	78.38%	91	114	79.82%	112	113	99.12%
	2	73	76	96.05%	89	100	89.00%	60	63	95.24%
	3	61	61	100.00%	49	97	50.52%	56	70	80.00%
	4	59	67	88.06%	117	156	75.00%	77	80	96.25%
	5	65	65	100.00%	40	50	80.00%	0	0	nd
	6	30	47	63.83%	41	48	85.42%	0	0	nd
	7	0	0	nd	86	124	69.35%	0	0	nd
	8	0	0	nd	54	81	66.67%	0	0	nd
	Total	346	390	88.72%	567	770	73.64%	305	326	93.56%
2012	1	48	48	100.00%	48	49	97.96%	57	60	95.00%
	2	56	56	100.00%	32	33	96.97%	65	66	98.48%
	3	59	59	100.00%	54	62	87.10%	44	55	80.00%
	4	56	57	98.25%	34	35	97.14%	46	47	97.87%
	5	0	0	nd	39	39	100.00%	0	0	nd
	6	0	0	nd	0	0	nd	0	0	nd
	7	0	0	nd	0	0	nd	0	0	nd
	8	0	0	nd	0	0	nd	0	0	nd
	Total	219	220	99.55%	207	218	94.95%	212	228	92.98%

**Table 3:** Results of the final GLMM models (using *p*-values as a selection criterion, dropping those variables with *p*-value > 0.05) for vole activity based on the indirect abundance index (IAI) and different variables potentially affecting vole abundance. Bold print indicates significant results. All 2-, 3-, and 4-way interactions not shown in the table were dropped during the modeling process.

Factor	Distance to Closest Unoccupied Nest-Box			Distance to Closest Occupied Nest-Box		
	χ^2^	df	*p*-Value	χ^2^	df	*p*-Value
Intercept	**39.11**	**1**	**<0.001**	**44.09**	**1**	**<0.001**
Distance	Dropped			**6.6**	**2**	**<0.05**
Year	0.94	1	>0.05	0.56	1	>0.05
Area	**23.58**	**2**	**<0.001**	**23.21**	**2**	**<0.001**
Season	**4.37**	**1**	**<0.05**	**4.49**	**1**	**<0.05**
Area:Season	**11.66**	**2**	**<0.05**	**11.66**	**2**	**<0.001**
Season:Year	**27.21**	**1**	**<0.001**	**26.95**	**1**	**<0.001**
Year:Area	**15.76**	**2**	**<0.001**	**17.45**	**2**	**<0.001**

**Table 4:** Results of the final GLMM models (using *p*-values as selection criteria, dropping those variables with *p*-value > 0.05) for vole activity based on the indirect abundance index (IAI) and different variables potentially affecting vole abundance. The number of fledglings indicates the total number of individuals sampled by quadrat in a circular buffer of 180 m or 540 m radius. Bold print indicates significant results. Dropped indicates that the variable was excluded from the analysis since its effect was not statistically significant. All 2-, 3- and 4- way interactions were dropped during the modeling process.

Factor	Number of Fledglings within 180 m			Number of Fledglings within 540 m		
	χ^2^	df	*p*-Value	χ^2^	df	*p*-Value
Fledglings	Dropped			**4.99**	**1**	**<0.05**
Year	**42.64**	**1**	**<0.001**	**43.74**	**1**	**<0.001**
Area	**20.65**	**2**	**<0.001**	**22.13**	**2**	**<0.001**
Year:Area	**16.08**	**2**	**<0.001**	**17.25**	**2**	**<0.001**

## Data Availability

The data presented in this study are available upon request from the corresponding author. The data are not publicly available due to privacy restrictions as it is being used in further studies for upcoming publications.

## Supplementary Materials

The following supporting information can be downloaded at: https://www.mdpi.com/article/10.3390/life13101963/s1: Table S1: Mean distances in meters between nest-boxes during the study period, all study areas pooled; Table S2: Results of the final GLMM models (using *p*-values as selection criterion, dropping those variables with *p*-value > 0.05) for vole activity based in the indirect abundance index (IAI) and different variables potentially affecting vole abundance, considering exclusively alfalfa fields; Table S3: Results of the final GLMM models (using *p*-values as selection criterion, dropping those variables with *p*-value > 0.05) for vole activity based on the indirect abundance index (IAI) and different variables potentially affecting vole abundance, considering all habitats except alfalfa fields; Figure S1: Study area of Villalar de los Comuneros (Valladolid province; VC); Figure S2: Study area of Boada de Campos/Capillas (Palencia province; BC); Figure S3: Study area of San Martín de Valderaduey (Zamora province; SMV); Figure S4: Boxplot of average number of fledglings in each study area and by habitat, after breeding season (summer season), considering a circular buffer of 180 m radius around every sampled quadrat, pooling years; Figure S5: Boxplot of average number of fledglings in each study area and by habitat, after breeding season (summer season), considering a circular buffer of 540 m radius around every sampled quadrat, pooling years; Figure S6: Common vole abundance (percentage of quadrats with presence) in alfalfa crops, considering distances to nest-boxes (occupied and unoccupied) and year, all study areas and seasons pooled, using an indirect abundance index (IAI); Figure S7: Common vole abundance (percentage of quadrats with presence) in alfalfa crops, considering distances to nest-boxes and season, all study areas and years pooled, using an indirect abundance index (IAI); Figure S8. Synthetic figure showing the average distance to occupied nest-boxes of sampled quadrats within alfalfa fields with absence (0) or presence (1), split by study area, year, and season; Figure S9. Common vole abundance (percentage of quadrats with presence) in all habitats except alfalfa crops, considering distances to nest-boxes and study areas, all seasons and years pooled, using an indirect abundance index (IAI); Figure S10. Synthetic figure showing the average distance of sampled quadrats within all habitats pooled, excluding alfalfa fields, with absence (0) or presence (1) of vole signs, split by study area, year, and season.
