# Supplementary material for: Local Effects of Nest-Boxes for Avian Predators over Common Vole Abundance during a Mid-Density Outbreak
Source: Life (Basel). 2023 Sep 26;13(10):1963. doi: 10.3390/life13101963 (PMC10608117; doi:10.3390/life13101963)
Supplement: Supplementary file 1 [file life-13-01963-s001.zip › life-2532235-supplementary.pdf]

Table S1. Mean distances in meters between nest-boxes during the study period, all study areas pooled. ALL indicates distance between all nest-boxes, ON indicates distance between occupied nest-boxes, UN indicates distance between unoccupied nest-boxes, and ON-UN distance between occupied and unoccupied nest-boxes.

Nest-box	Year		Mean
	2011	2012	
ALL	249.7	245.3	247.5
ON	442.6	332	387.3
UN	309.7	375.7	342.7
ON-UN	351.8	392.4	372.1

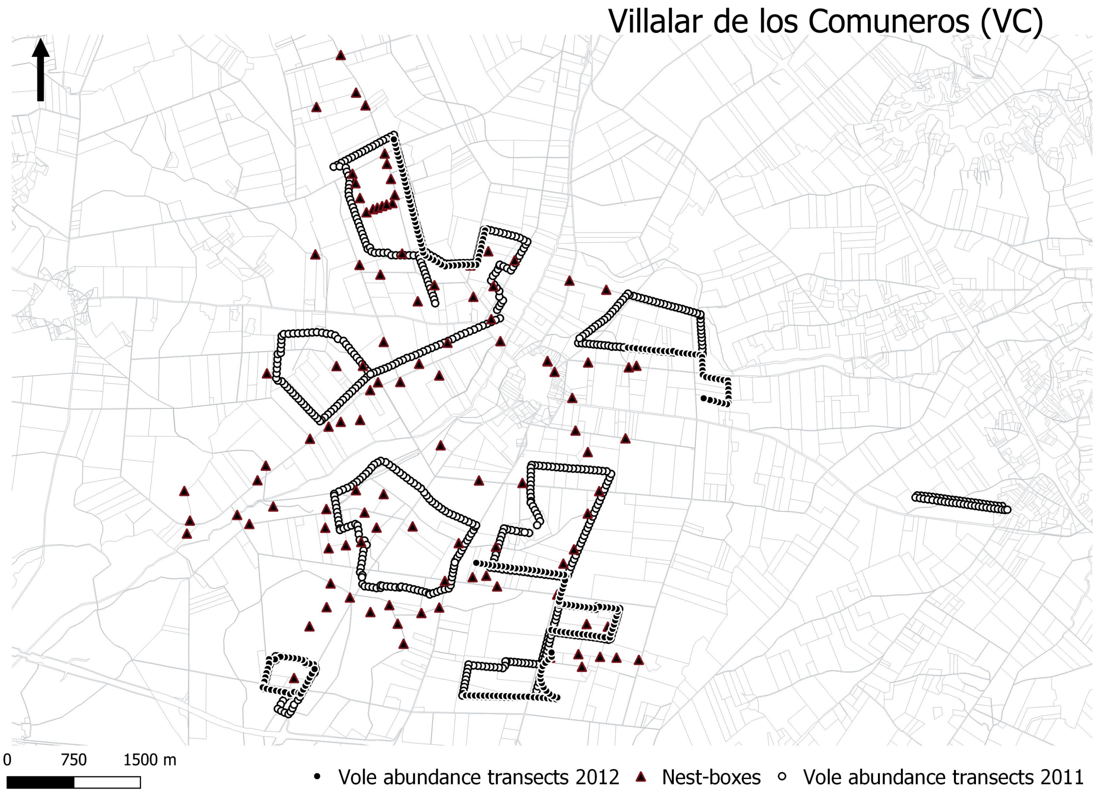

Figure S1. Study area of Villalar de los Comuneros (Valladolid province; VC). The points represent the sampling squares made to estimate abundances of voles through indirect indices. The filled triangles are the location of the nest boxes.

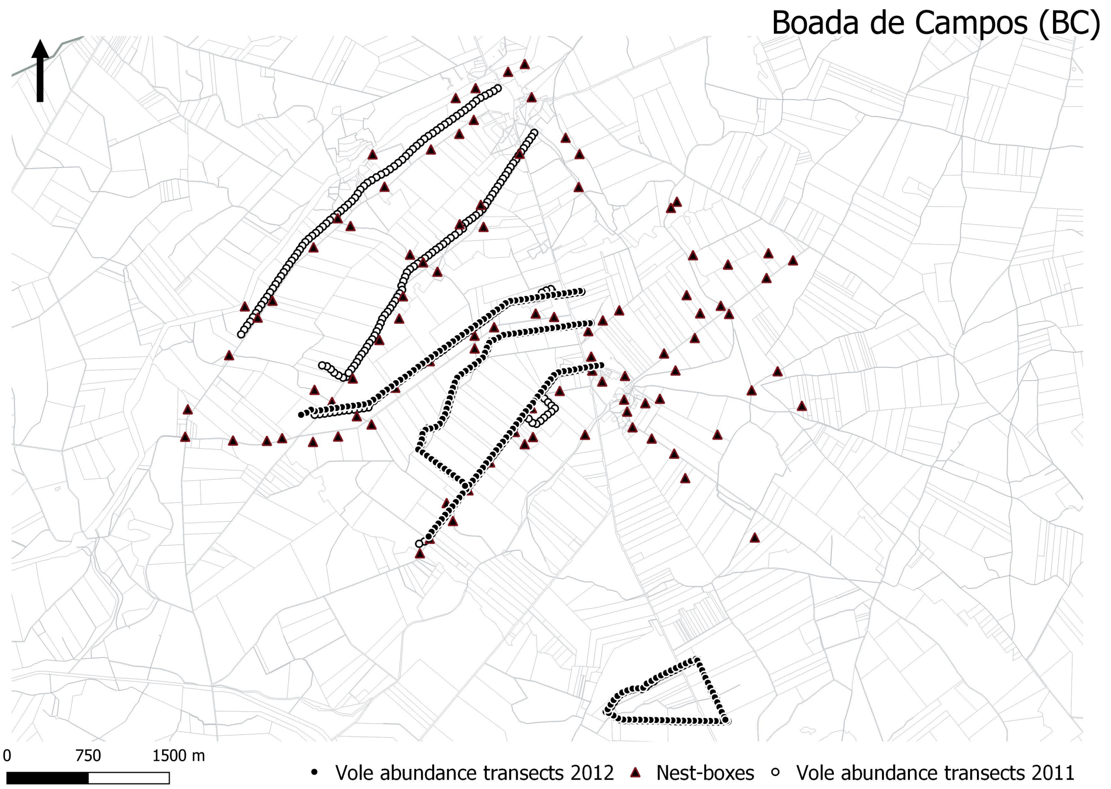

Figure S2. Study area of Boada de Campos / Capillas (Palencia province; BC). The points represent the sampling squares made to estimate abundances of voles through indirect indices. The filled triangles are the location of the nest boxes.

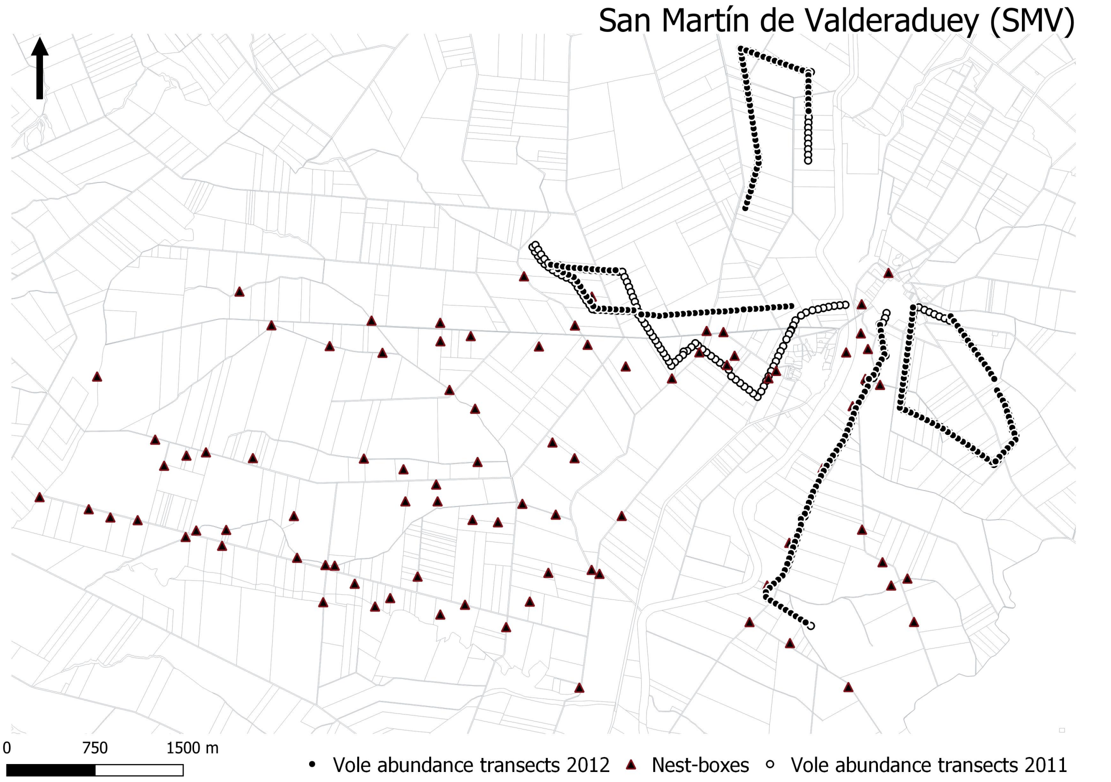

Figure S3. Study area of San Martín de Valderaduey (Zamora province; SMV). The points represent the sampling squares made to estimate abundances of voles through indirect indices. The filled triangles are the location of the nest boxes.

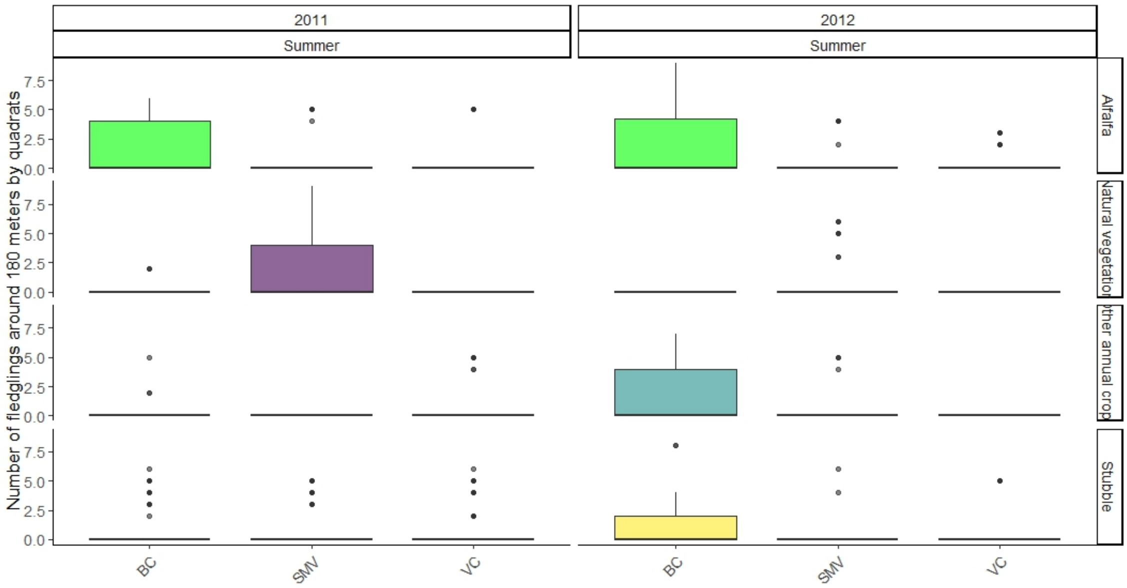

Figure S4. Boxplot of average number of fledglings in each study area and by habitat, after breeding season (summer season), considering a circular buffer of 180 meters radius around every sampled quadrat, pooling years. Abbreviations for study areas as in Figure 1. "●" represents outliers.

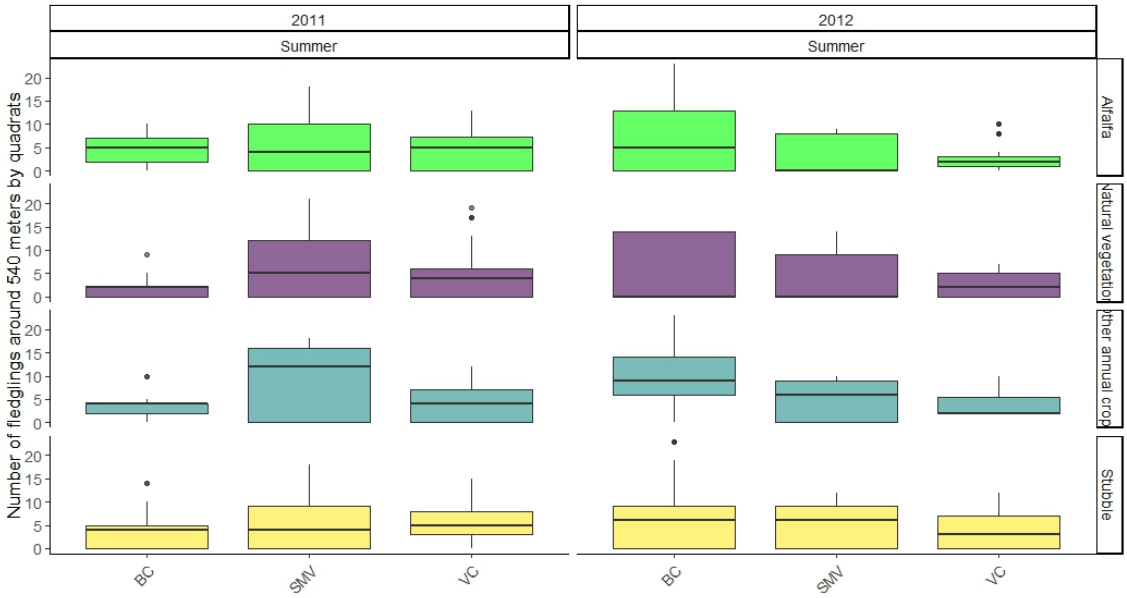

Figure S5. Boxplot of average number of fledglings in each study area and by habitat, after breeding season (summer season), considering a circular buffer of 540 meters radius around every sampled quadrat, pooling years. Abbreviations for study areas as in Figure 1. "●" represents outliers.

Table S2. Results of the final GLMM models (using p-values as selection criterion, dropping those variables with p-value > 0.05) for vole activity based in the indirect abundance index (IAI) and different variables potentially affecting vole abundance, considering exclusively alfalfa fields. Bold print indicates significant results. All 2-, 3- and 4-way interactions not shown in the table were dropped during the modeling process.

Factor	Distance to Closest Occupied Nest-box			Distance to Closest Unoccupied Nest-box		
	χ^2	df	p-value	χ^2	df	p-value
Distance	Dropped			**19.54**	**5**	**< 0.01**
Year	**15.004**	**2**	**< 0.001**	**20.52**	**4**	**< 0.001**
Area	**14.47**	**4**	**< 0.01**	**11.68**	**4**	**< 0.05**
Season	**27.74**	**4**	**< 0.001**	**36.94**	**6**	**< 0.001**
Area:Season	**14.97**	**4**	**< 0.01**	**9.66**	**2**	**< 0.01**
Season:Year	**6.95**	**2**	**< 0.05**	**13.9**	**3**	**< 0.01**
Distance:Year	Dropped			**7.03**	**2**	**< 0.05**
Distance:Season	Dropped			**9.92**	**2**	**< 0.01**

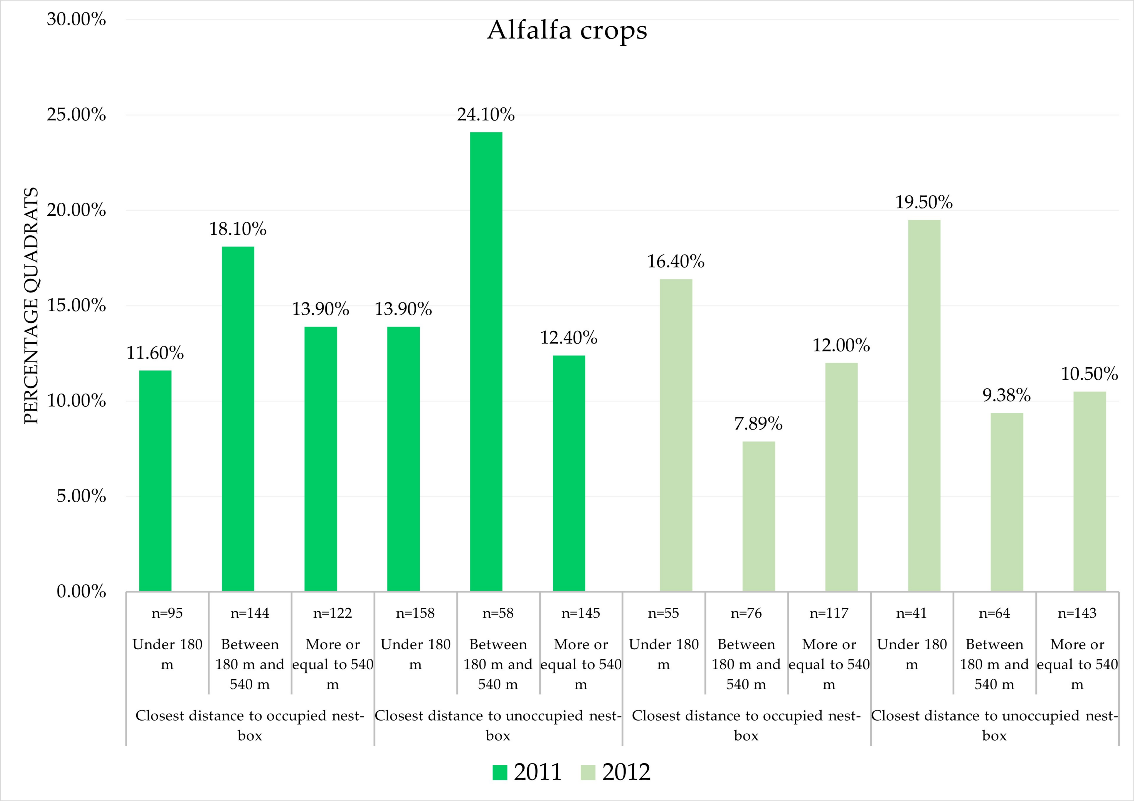

Figure S6. Common vole abundance (percentage of quadrats with presence) in alfalfa crops, considering distances to nest-boxes (occupied and unoccupied) and year, all study areas and seasons pooled, using an indirect abundance index (IAI). Under each year appears the number of sampled quadrats per distance category and year (n).

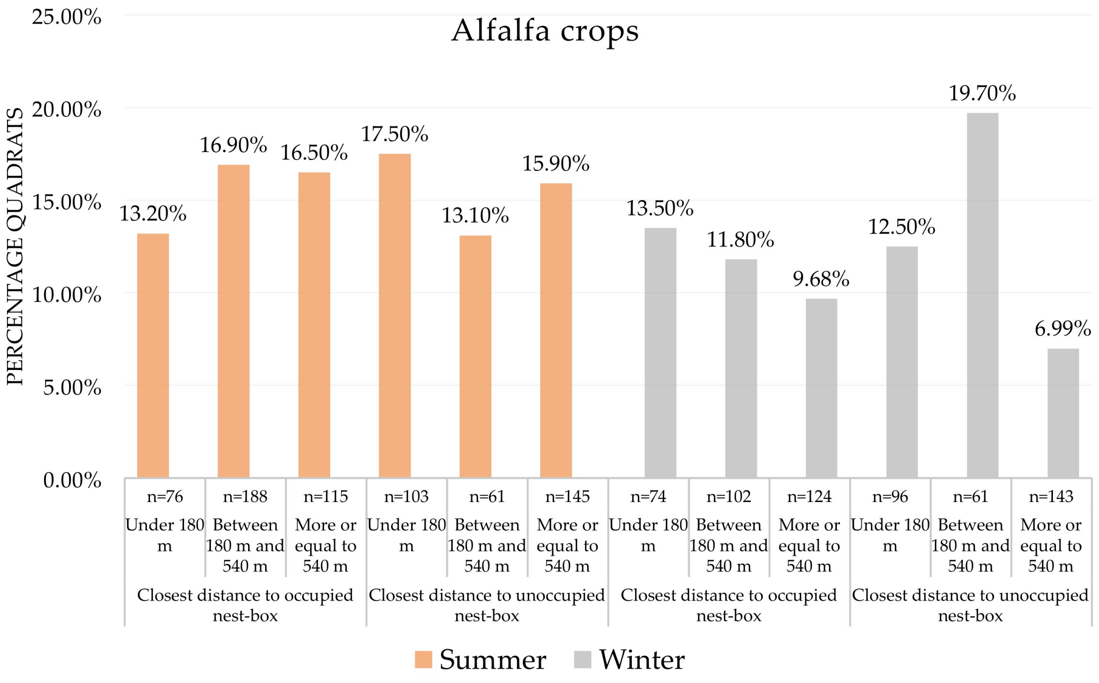

Figure S7. Common vole abundance (percentage of quadrats with presence) in alfalfa crops, considering distances to nest-boxes and season, all study areas and years pooled, using an indirect abundance index (IAI). Under each year appears the number of sampled quadrats per distance category and season (n).

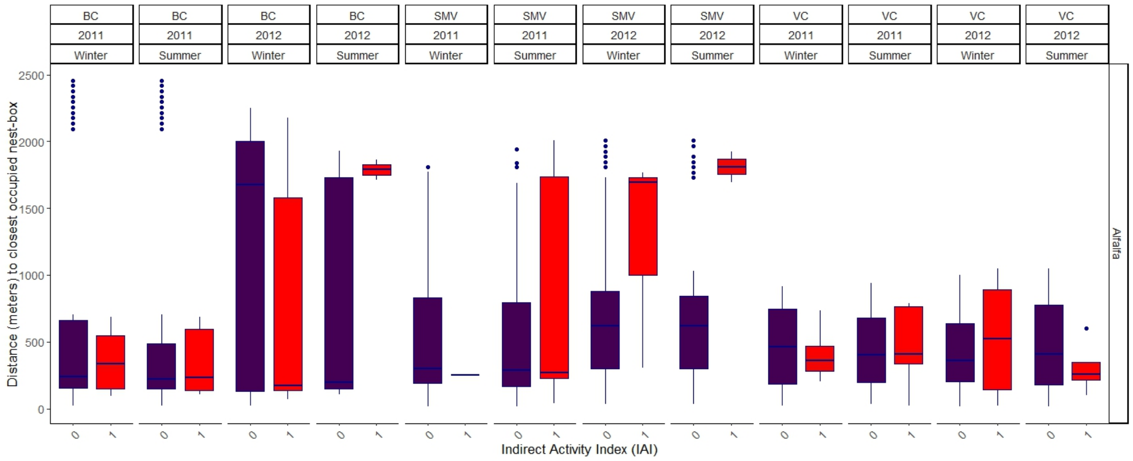

Figure S8. Synthetic figure showing average distance to occupied nest-boxes of sampled quadrats within alfalfa fields with absence (0, black bars) or presence (1, red bars), split by study area, year and season. Abbreviations for study areas as in Figure 1.

Table S3. Results of the final GLMM models (using p-values as selection criterion, dropping those variables with p-value > 0.05) for vole activity based in the indirect abundance index (IAI) and different variables potentially affecting vole abundance, considering all habitats except alfalfa fields. Bold print indicates significant results. All 2-, 3- and 4-way interactions not shown in the table were dropped during the modeling process.

Factor	Distance to Closest Occupied Nest-box			Distance to Closest Unoccupied Nest-box		
	χ^2	df	p-value	χ^2	df	p-value
Distance	**29.59**	**6**	**< 0.001**	Dropped		
Year	**71.74**	**4**	**< 0.001**	**67.74**	**4**	**< 0.001**
Area	**56.06**	**8**	**< 0.001**	**44.99**	**4**	**< 0.001**
Season	**32.47**	**2**	**< 0.001**	**33.45**	**2**	**< 0.001**
Area:Season	Dropped			Dropped		
Season:Year	**36.12**	**2**	**< 0.001**	**26.71**	**1**	**< 0.001**
Year:Area	**24.29**	**3**	**< 0.001**	**18.5**	**2**	**< 0.001**
Distance:Area	**9.05**	**2**	**< 0.05**	Dropped		

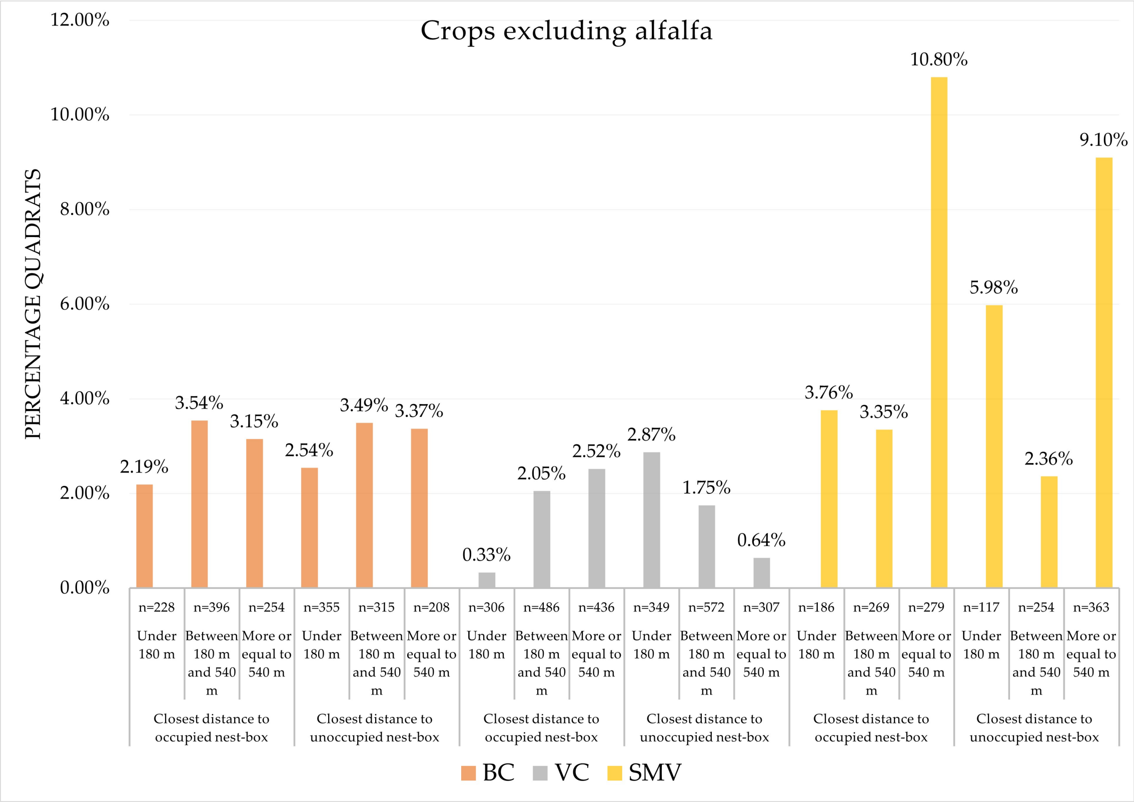

Figure S9. Common vole abundance (percentage of quadrats with presence) in all habitats except alfalfa crops, considering distances to nest-boxes and study areas, all seasons and years pooled, using an indirect abundance index (IAI). Under each year appears the number of sampled quadrats per distance category and area (n). Abbreviations for study areas as in Fig. 1.

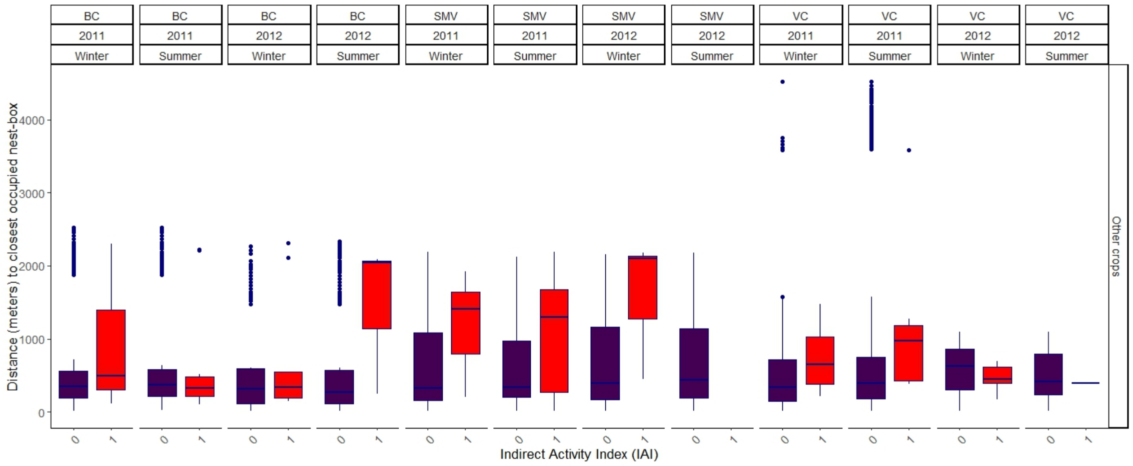

Figure S10. Synthetic figure showing average distance of sampled quadrats within all habitats pooled, excluding alfalfa fields, with absence (0, black bars) or presence (1, red bars) of vole signs, split by study area, year and season. Abbreviations for study areas as in Figure 1.
